# Experiences of Primiparous Mothers Using ChatGPTfor Breastfeeding and Infant Care

**DOI:** 10.1111/mcn.70236

**Published:** 2026-07-28

**Authors:** Eyşan Hanzade Savaş, Maide Nur Tümkaya, Ezgi Hasret Kozan Çıkırıkçı, Pelin Gökoğlu Gürer, Nevra Didem Kılınç

**Affiliations:** ^1^ Pediatric Oncology Department Regina Margherita Children's Hospital Turin Italy; ^2^ Department of Nursing İstanbul Atlas University Istanbul Türkiye; ^3^ Koç University School of Nursing Istanbul Türkiye; ^4^ Department of Child Development Haliç University Istanbul Türkiye; ^5^ Department of Nursing İstanbul Arel University Istanbul Türkiye

**Keywords:** artificial intelligence, breastfeeding, infant care, postpartum period, primiparous mothers

## Abstract

Artificial intelligence‐based conversational tools such as ChatGPT are increasingly used by mothers to obtain health‐related information during early motherhood. This study aimed to explore and describe the experiences and perceptions of primiparous mothers using ChatGPT as a source of information for breastfeeding and early infant care. A qualitative descriptive study was conducted. Fifteen primiparous mothers in Türkiye were recruited using purposive sampling from a pediatric outpatient clinic. Data were collected through semi‐structured, in‐depth online interviews and analyzed using Braun and Clarke's reflexive thematic analysis. The study followed the COREQ checklist to enhance methodological transparency. A total of 15 primiparous mothers participated in the study (28.7 ± 2.5 years). The mean age of infants was 4.2 ± 1.6 months, and most were female (73.3%). All participants reported regular internet access and used smartphones to access ChatGPT, with use ranging from daily to a few times per month. Three main themes emerged: (1) ChatGPT as an accessible alternative information source in early motherhood; (2) the role of ChatGPT in reducing uncertainty in early motherhood, characterized by support in unfamiliar situations, reassurance during moments of concern, and strengthening decision‐making skills and maternal confidence; and (3) rare use of ChatGPT, reflecting mothers' deliberate avoidance of the tool in serious or complex infant health situations and their preference for personalized professional support. ChatGPT may serve as a complementary information resource during early motherhood, offering reassurance and confidence for everyday care; however, it cannot replace individualized professional healthcare support.

## Summary

- Primiparous mothers increasingly utilize ChatGPT as a 24/7 alternative to professional support for routine breastfeeding and infant care due to perceived barriers in accessing healthcare providers.
- Beyond information retrieval, AI provides significant psychosocial support by offering non‐judgmental reassurance, which helps reduce maternal concern and strengthens caregiving confidence.
- While mothers instinctively defer to medical professionals for complex clinical issues, the reliance on AI for routine care highlights a critical need for structured, accessible postpartum telehealth services.

## 1 Introduction

The transition from the hospital environment to the home setting with a newborn may represent a dynamic and comprehensive period of adjustment for mothers (Lorén et al. 2024; Hjälmhult and Lomborg 2012). Early motherhood is often characterized by increased sensitivity; during this time, mothers may find themselves adapting to new responsibilities, experiencing moments of uncertainty, and gradually becoming accustomed to the way newborn care reshapes everyday life (Simmons 2020). Sudden changes in daily routines, the constant needs of the infant, and the mother's own physical recovery process may make this transition physically and psychosocially demanding (Lorén et al. 2024). Therefore, mothers' information needs increase during this period; however, these needs extend beyond technical knowledge to include reassurance, validation, and an internal sense of confidence that they are doing the right things (Nan et al. 2020). Many mothers experience uncertainty in the early stages of motherhood regarding the frequency and duration of breastfeeding, whether the baby is getting enough nutrition (Savaş et al. 2024), whether weight gain is normal, and other care needs (Türkmen et al. 2022; Salarvand et al. 2020; Borrelli et al. 2018). Therefore, the responsibilities of interpreting infant behavior and handling daily care tasks in the early stages of motherhood can sometimes cause anxiety in mothers (Petzoldt et al. 2016; Türkmen et al. 2022).

In early motherhood, providing appropriate support and education plays a critical role in facilitating adjustment to maternal and infant care. Various factors influence this adjustment, including maternal self‐efficacy, access to social support, availability of healthcare professionals, and clear pathways to reliable information (Tran et al. 2023; Sharifipour et al. 2023; Asadi et al. 2020). However, advice from the environment and prevailing social expectations can hinder mothers' efforts to navigate caregiving decisions Lorén et al. 2024). Additionally, when professional support is limited, primiparous mothers may have to cope with uncertainties on their own, which can increase anxiety and undermine their confidence in their caregiving roles (Nan et al. 2020). Thus, inadequate support, fragmented information sources, and feelings of isolation are widely recognized as key barriers to adjusting to effective maternal and infant care, making the early motherhood experience more fragile (Lorén et al. 2024; Sharifipour et al. 2023).

In recent years, increased access to digital information sources has substantially transformed how individuals seek health and care‐related information. Web‐based platforms, mobile applications, and online support tools have become increasingly popular resources, offering rapid and continuous access to information, particularly for everyday or urgent caregiving concerns (Al Shboul et al. 2024). Alongside the development of artificial intelligence‐based conversational tools, systems such as ChatGPT have increasingly been used for health‐related topics, including breastfeeding and early infant care (Kerimoglu Yildiz et al. 2025; Dol et al. 2022). The literature indicates that mothers increasingly use digital tools primarily because they offer quick and easily accessible information (Özdemir Kaçer 2025; Dol et al. 2022), a trend that is particularly pronounced among millennial mothers, who constitute most women of childbearing age today (Hikmah 2025). Nevertheless, the literature also highlights several limitations associated with the use of AI‐based conversational tools in healthcare. indicates that while such tools are perceived as practical and accessible for basic health‐related questions, they raise concerns regarding reliability and accuracy (Özdemir Kaçer 2025; Molu 2025; Shboul et al. 2024). The growing use of AI‐based tools by mothers, together with the known limitations of these technologies, underscores the need for a deeper understanding of these experiences during early motherhood (Agudelo‐Pérez et al. 2024). Accordingly, this study aims to explore and describe how primiparous mothers use ChatGPT as a source of health information for breastfeeding and early infant care.

## 2 Methods

### 2.1 Design

A qualitative descriptive study using reflexive thematic analysis was conducted to gain an in‐depth understanding of primiparous mothers' perspectives on using ChatGPT as a source of health information during early motherhood. This approach was considered appropriate for the study's aim of exploring how primiparous mothers use ChatGPT, how they perceive the information obtained, and how this tool fits into their everyday practices related to breastfeeding and early infant care. Reflexive thematic analysis, as described by Clarke and Braun (2017), enabled a flexible and systematic examination of patterns of meaning across the dataset while acknowledging the active role of the researchers in the analytic process. The study adhered to the Consolidated Criteria for Reporting Qualitative Research (COREQ) checklist to enhance transparency and rigor in the reporting of qualitative research (Tong et al. 2007).

### 2.2 Setting

This study was conducted in the pediatric outpatient clinic of a university hospital in Istanbul, Türkiye. Participants were recruited among primiparous mothers who attended the clinic either for their infants' routine neonatal check‐up in the first week after birth or for the second or sixth‐month vaccination visits.

### 2.3 Sampling

Criterion‐based purposive sampling was used to recruit a homogeneous sample of primiparous mothers and to obtain rich data relevant to the study aim. Inclusion criteria were (a) being a primiparous mother, (b) having an infant younger than 6 months, (c) having used ChatGPT at least once for breastfeeding‐ or infant care‐related questions, (d) residing in Türkiye, and (e) voluntarily agreeing to participate in the study. Exclusion criteria included (a) mothers or infants with a diagnosed chronic illness, (b) infants admitted to a neonatal intensive care unit (NICU), and (c) mothers who experienced serious postpartum complications. In line with Saunders et al. (2018), saturation was not treated as a generic endpoint but as a context‐specific judgment based on the redundancy of codes, recurrence of patterns, and adequacy of the data in addressing the research aim. Saturation was assessed iteratively: after each interview, transcripts were read and preliminarily coded, and author team discussed whether emerging accounts contributed new analytic insights. From the thirteenth interview onwards, participants' accounts increasingly reflected patterns already represented in the dataset; two interviews were conducted to confirm this, and no new codes or thematic dimensions emerged. Data saturation was achieved after interviewing 15 primiparous mothers.

### 2.4 Data Collection

Data were collected between 8 October and 20 December 2025 through semi‐structured, in‐depth individual interviews. Participants were recruited from a university hospital during routine infant follow‐up visits, including healthy‐baby check‐ups and vaccination appointments. After the clinical visit, eligible mothers were approached in person, informed about the purpose and procedures of the study, and invited to participate by one of the researchers. Written informed consent was obtained, and eligibility criteria were assessed during this initial meeting. Following consent, interviews were scheduled at a mutually convenient time and conducted online via Zoom. Two data collection tools were used: a Demographic Information Form and a Semi‐Structured Interview Guide. Participants completed the demographic form via a secure online link at the start of each Zoom session.

The *Demographic Information Form* was developed by the researchers and consisted of nine questions collecting information on participants' sociodemographic characteristics, obstetric background, and technology‐use characteristics related to ChatGPT.

Subsequently, all interviews were conducted by the first author, who has experience in qualitative research, holds a PhD degree in pediatric nursing, and had no prior relationship with the participants. Each participant took part in a single interview session lasting approximately 30–45 min. All interviews were conducted in Turkish and audio‐recorded with participants' permission; no video recordings were made.

The interviews were guided by an open‐ended semi‐structured interview guide developed based on a comprehensive review of the relevant literature and expert consultation. The interview guide was designed to explore how primiparous mothers use ChatGPT as a source of health information during early motherhood, their views on the information obtained, and the role of this tool in their everyday practices related to breastfeeding and early infant care. The guide consisted of four open‐ended questions (Table 1). The guide consisted of four broad open‐ended questions (Table 1). Additional probing and follow‐up questions were used to encourage participants to elaborate on their experiences and clarify emerging issues. Examples included “Can you tell me more about that experience?,” “What made you decide to use ChatGPT in that situation?,” “How did you feel after receiving that information?,” “Can you give an example?,” and “How did ChatGPT's response influence what you did next?.” These prompts were adapted according to participants' responses and facilitated an in‐depth exploration of their experiences.

**Table 1:** Interview guide.

Interview question	Purpose
How did you first start using ChatGPT for breastfeeding or infant care?	To explore initial motivations and pathways for using ChatGPT in early motherhood
What kinds of questions did you ask ChatGPT about your baby?	To identify the types of infant care and breastfeeding‐related concerns addressed through ChatGPT
When did you feel the need to use ChatGPT the most?	To examine situations or contexts prompting ChatGPT use
How did ChatGPT's information support you in breastfeeding or infant care?	To understand the perceived supportive role of ChatGPT in caregiving practices

### 2.5 Data Analysis

Descriptive statistics were used to summarize participants' sociodemographic and technology‐use characteristics, including frequencies, percentages, means, and standard deviations.

Qualitative interview data were analyzed using reflexive thematic analysis, drawing on the approach articulated by Braun and Clarke (2006) and further elaborated by Clarke and Braun (2017). Analysis proceeded through six recursive and iterative phases: familiarization with the data through repeated reading of transcripts; generation of initial codes; searching for patterns of shared meaning; reviewing and refining candidate themes; defining and naming themes; and producing the final analytic report supported by illustrative data extracts.

All interviews were transcribed verbatim by the research team, and MAXQDA software (version 2020.2.2) was used to support data management and organization. Analysis was led by the principal researcher, who engaged in close, repeated interaction with the data throughout the analytic process. Other members of the research team contributed through ongoing reflexive discussions, in which emerging interpretations, patterns of meaning, and analytic decisions were critically examined and refined. Rather than seeking coding consensus, these discussions were used to enhance reflexivity, deepen interpretation, and ensure analytic rigor.

All authors contributed to the interpretation of the findings and the finalization of themes, and the written report.

Interviews were conducted in Turkish. Selected quotations were translated into English using a two‐step process involving initial translation by a researcher familiar with the study context, followed by independent review by another team member to ensure linguistic accuracy and cultural relevance. Any discrepancies were resolved through discussion. To protect confidentiality, each participant was assigned a unique identifier (e.g., P1, P2, P3), which was used consistently throughout the findings.

### 2.6 Rigor and Trustworthiness

This study applied Yardley's principles of context sensitivity, rigor, transparency, and impact (Yardley 2000). To ensure context sensitivity, direct quotations were used to allow participants to have their voices heard and to allow readers to follow the interpretation process. Rigor was enhanced through an iterative analytical process; initial coding and theme development were performed by the first author and subsequently reviewed and discussed with co‐authors (MNT, PGG). Transparency was ensured by clearly documenting each stage of the research process and analytical decisions. Reflectivity was maintained throughout the study. The first author is a pediatric nurse with clinical experience in neonatal intensive care and maternal‐infant units, where she has supported mothers in breastfeeding and maternal‐infant adaptation. She also has research experience in breastfeeding and maternal‐child health. The research team acknowledged that their professional backgrounds and previous interests in breastfeeding support, as well as emerging AI‐based information tools, could shape the interpretation of the data. To address this issue, researchers engaged in continuous discussions during data analysis, critically evaluating their assumptions and actively considering alternative interpretations. This process helped ensure that the findings were based on participants' accounts rather than the researchers' biases.

### 2.7 Artificial Intelligence Disclosure

**Figure 1:**
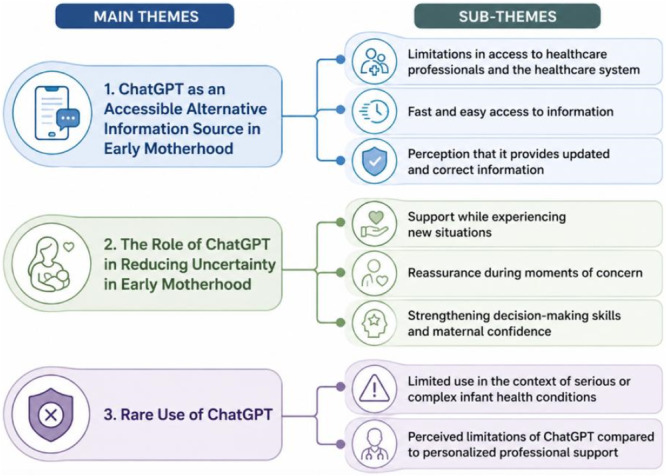
Main themes and subthemes.

Figure 1 was generated using ChatGPT (OpenAI) based on prompts developed by the authors. The generated output was reviewed and validated by the authors, who assume full responsibility for the final content.

### 2.8 Ethics Statement

Ethical approval was obtained from the University Social Sciences Ethics Committee (approval number 2025.372.IRB3.141), and institutional permission was obtained from the healthcare institution on October 7, 2025. The study was conducted in accordance with the ethical principles of the Declaration of Helsinki.

## 3 Results

A total of 15 primiparous mothers participated in the study. The mean age of the participants was 28.73 ± 2.46 years (min: 25, max: 34). Most mothers had completed university‐level education (73.3%). The majority of participants were employed (60.0%), while others were unemployed or on maternity leave. In terms of household income, most mothers reported that their income was equal to their expenses (66.7%). All participants were primiparous. Regarding the type of delivery, 66.7% of the mothers reported having a cesarean section, while 33.3% reported a vaginal birth. The mean age of the infants was 4.2 ± 1.56 months, and 73.3% were female. All mothers reported having regular internet access at home. Smartphones were the primary devices used to access ChatGPT by all participants. The frequency of ChatGPT use varied, with 40.0% of mothers reporting daily use and 40.0% reporting use a few times a week, while 20.0% reported using ChatGPT a few times a month. The detailed characteristics of the participants are presented in Table 2.

**Table 2:** Characteristics of the participants.

Characteristics	Mean ± SD	Min‐max
Age of primiparous mother (years)	28.73 ± 2.46	25–34
Infant's current age (months)	4.2 ± 1.56	2–6

	***n***	**%**
Marital status		
Married	15	100
Education level		
High school	2	13.3
University	11	73.3
Postgraduate	2	13.3
Employment status		
Employed	9	60.0
Unemployed	4	6.7
On maternity leave	2	13.3
Income Status		
Income equals expenses	10	66.7
Income slightly exceeds expenses	4	26.7
Income is less than expenses	1	6.7
Type of delivery		
Vaginal birth	5	33.3
Cesarean section	10	66.7
Infant's gender		
Female	11	73.3
Male	4	26.7
Regular internet access at home		
Yes	15	100
Primary device used to access ChatGPT		
Smartphone	15	100
Frequency of ChatGPT use		
Daily	6	40.0
A few times a week	5	33.3
A few times a month	2	13.3
Rarely	2	13.3

The findings of this qualitative study revealed that primiparous mothers described ChatGPT as a supportive resource during the early stages of motherhood, while also highlighting situations in which its use was limited or unnecessary. Data analysis identified three main themes reflecting mothers' experiences with ChatGPT use. The first main theme, ChatGPT as an Accessible Alternative Information Source in Early Motherhood, included three sub‐themes: (i) limitations in access to healthcare professionals and the healthcare system, (ii) fast and easy access to information, and (iii) the perception that ChatGPT provides updated and correct information. The second main theme, The Role of ChatGPT in Reducing Uncertainty in Early Motherhood, comprised three sub‐themes: (i) support while experiencing new situations, (ii) reassurance during moments of concern, and (iii) strengthening decision‐making skills and maternal confidence. The third main theme, Rare Use of ChatGPT, reflected mothers' deliberate decisions to restrict or avoid ChatGPT use and included two sub‐themes: (i) limited use in the context of serious or complex infant health conditions, and (ii) perceived limitations of ChatGPT compared to personalized professional support. The main themes and sub‐themes are presented in Figure 1.

### 3.1 Theme 1: ChatGPT as an Accessible Alternative Information Source in Early Motherhood

Our findings revealed that primiparous mothers described ChatGPT as a readily available resource that they integrated into their daily caregiving practices during early motherhood. It was observed that the need for rapid access to information, concerns about the cost of professional support, and difficulties in reaching healthcare professionals shaped mothers' use of ChatGPT.

*Limitations in Access to Healthcare Professionals and the Healthcare System:* One of the main reasons for the widespread use of ChatGPT was mothers expressed concern about “not wanting to disturb physicians too frequently.” Many mothers reported that they felt unable to contact physicians for what they perceived as unnecessary issues, while at the same time describing a strong need to ask frequent questions due to this being their first experience with newborn care. ‘’ There's no time limit to how often you can reach ChatGPT. You can get a response at any moment, overnight, or early in the morning. For me, it was always readily available.” (P7)

Mothers also reported that a lack of feedback from healthcare professionals and uncertainty about whom to contact contributed to their use of ChatGPT. One mother stated:

Who can I keep calling all the time? There are people around me, but I do not want to keep asking them either. Especially in the first days after discharge, I really needed a professional who could support us, either in person or by phone. However, because there was no such person or centre, using ChatGPT during those days was something I can say saved my life.

(P12)

*Fast and Easy Access to Information:* Primipara mothers reported that the use of ChatGPT was common primarily due to its easy accessibility. All mothers who used ChatGPT during newborn care indicated that they frequently preferred it as a source of information because it allowed rapid access to information without waiting. One mother described this as:

When we first arrived home from the hospital, we didn't know what to do. We'd received training there, but we were so excited that we couldn't remember anything. The moment my baby fell asleep, I would ask ChatGPT all the questions I had. In the first few days and afterwards, since there was no one else with us, my wife and I tried to understand and solve our baby's sleep problems and difficulties. ChatGPT had become like a member of our family.

(P8)

Another mother reported using ChatGPT through voice messages, which allowed her to avoid lengthy typing and to seek information even while breastfeeding at night. She explained that this feature was particularly helpful during times when calling someone was not feasible or when others were unavailable, stating:

I didn't have the time to write long messages or make phone calls at night. As mothers, we know that it's not always possible to talk on the phone or reach someone when you need to. With ChatGPT, I could even ask questions using voice recordings.

(P11)

*Perception of providing up‐to‐date and accurate information:* Many mothers believed that ChatGPT provided them with cumulative answers by scanning and synthesizing information found on the internet. They stated that this saved them time because they didn't need to search multiple websites individually, and they felt ChatGPT did the searching for them. One mother expressed it this way:

I didn't have time to search for information by browsing many sites. ChatGPT reviewed all the data on the internet and reported a cumulative result. Therefore, I preferred it because it made my job much easier.

(P1)

In addition, some mothers reported viewing ChatGPT positively because they perceived it as up‐to‐date and as a technological tool. Mothers described feeling that the experiences of people around them were outdated and stated that they could not rely on advice from relatives or close contacts, as practices and information had changed rapidly. One mother explained:

When I gave birth, there were many people around me, but the youngest among them had a child who was thirty years old. Their comments and practices belonged to thirty years ago. Everything has changed so much now that ChatGPT feels more technological and up to date for me. I trusted it more than the people around me.

(P2)

### 3.2 Theme 2: The Role of ChatGPT in Reducing Uncertainty in Early Motherhood

Mothers described ChatGPT as a source of reassurance when encountering unfamiliar situations related to breastfeeding and infant care. Participants reported that receiving timely information and encouragement helped them feel more comfortable managing situations they perceived as uncertain or concerning. In addition, some mothers perceived that having access to information supported their decision‐making and contributed to a greater sense of confidence in their parenting role. This theme included three sub‐themes: support while experiencing new situations, reassurance during moments of concern, and strengthening decision‐making skills and maternal confidence.

*Support while experiencing new situations:* Some mothers reported developing practical strategies for using ChatGPT during newborn care. Several mothers noted that they were aware of the importance of monitoring weight gain in the early days after birth but found it difficult to keep track of feeding routines due to fatigue and busy days. One mother described using ChatGPT for this purpose, stating:

It created a daily feeding and elimination log for me based on the information I shared via voice messages. At the end of the day, it summarized feeding duration and diaper counts, which helped me monitor feeding routines more carefully during the early days.

(P4)

Several mothers also reported that the ability to send photographs was a significant convenience for them. Mothers described sending photographs to ChatGPT during the umbilical cord separation period to assess whether the process was progressing normally. One mother stated:

We were warned about the umbilical cord, such as preventing infection and folding the diaper properly. However, we did not know how to clean it when it became dirty, and its colour changed during the day. Every day, it looked different to me and became a source of concern. I sent a photo to ChatGPT every day and asked whether it looked normal. This was not something I could do with other search engines.

(P15)

Another mother also stated that:

We were told that mucus in the stool was important and that we should monitor it, but I did not know what mucus actually was. When I felt worried, I started sending photos of the stool to ChatGPT, and when it evaluated them, I felt reassured.

(P12)

Some mothers also reported frequent use of ChatGPT for issues such as sleep routines, vomiting, and reflux. These experiences were described as new and unfamiliar, and mothers reported uncertainty about how to manage them. One mother described this as follows:

In the beginning, it was a process where I could not understand whether my baby was hungry or full, or whether they were sleepy or not. Then I started managing everything with ChatGPT. For example, I asked whether a full baby vomits, and it explained what vomit looks like depending on whether it is digested or not, how often a full baby should have bowel movements, and what sleep patterns should be like. And it worked for us.

(P14)

*Reassurance during moments of concern:* Mothers reported that interactions with ChatGPT sometimes provided reassurance during periods of uncertainty. Many mothers reported that ChatGPT adopted a calming approach when they felt distressed about certain issues. One mother, whose infant experienced weight loss and who had been informed that formula feeding or a short hospital stay might be necessary, described her experience as follows:

At the hospital, they told me that if I couldn't feed my baby adequately, we might need to use formula, or my baby could stay in intensive care. I tried very hard but still felt unsure, and my baby was crying constantly. I used ChatGPT to create a feeding schedule; it followed the process and was always encouraging. It kept saying things like ‘You can do it’ or ‘Good job, you're almost there,’ almost as if there was a daily goal. It also showed me how much my baby was getting on average. It was supportive and helped calm me down, especially since everyone around me was very stressed and only told me to breastfeed.

(P4)

In addition, some mothers emphasized the nonjudgmental nature of ChatGPT, which made them feel more comfortable seeking support. One mother stated:

Honestly, ChatGPT even provided psychological support during the postpartum period. Whenever you ask a question, they always offer support. They do this without judgment; sometimes, when I ask questions to people around me, they look at me strangely. I felt more comfortable asking questions on ChatGPT.

(P10)

Another mother highlighted the contrast between ChatGPT and conventional search engines, describing ChatGPT as providing a calmer and more reassuring interaction:

When I search for something using other common search engines, many negative outcomes appear and make me anxious. However, ChatGPT takes a much calmer approach and does not create panic.

(P4)

*Strengthening Decision‐Making Skills and Maternal Confidence:* Many mothers who participated in the study reported that using ChatGPT provided them with a sense of autonomy in managing early motherhood. One mother stated:

I constantly needed to ask something from everyone around me, and I was not satisfied. Everyone's experience is different. Thanks to ChatGPT, I started handling some issues on my own.

(P13)

Another mother summarized her experience as follows:

During that period, I could not accept wanting to ask everything. I was afraid of whether I was incapable or whether people would judge me. Being able to handle simple things on my own by asking ChatGPT gave me a sense of autonomy and freedom. I started to feel competent and moved away from the feeling of being dependent on others.

(P4)

### 3.3 Theme 3: Rare Use of ChatGPT

Findings related to the final theme indicated that some primiparous mothers reported selective or limited use of ChatGPT during early motherhood. In situations involving significant clinical concerns, some mothers preferred to consult directly with physicians.

*Limited use in the context of serious or complex infant health conditions:* Mothers whose babies have some health problems have indicated that they do not prefer using ChatGPT. In these cases, mothers expressed concerns about relying on AI‐generated information for high‐risk situations or those requiring close medical supervision. One mother, whose baby developed allergies from the first month, described her limited use of ChatGPT for baby care with the following words:

From the very first month, we had a serious allergy problem. I had to constantly monitor her stool and be careful about what I ate. Each time, the severity of the allergy increased, and we had to go to the emergency room. We never had a situation where I could take any risks. Whatever I ate, I would encounter a problem when I breastfed my baby the next day, so I couldn't trust information from the internet. I had to get an expert opinion on every matter.

(P3)

Similarly, other mothers have indicated that they consult ChatGPT for general information, but prefer to see healthcare professionals when symptoms appear severe, persistent, or potentially harmful to their babies.

If it were something simple, such as sleep or feeding routines, I might ask ChatGPT. However, if my baby had a fever or something that genuinely worried me, I would contact our doctor directly because I wanted advice specific to my baby. ChatGPT generally provides similar responses to everyone, but those responses may not reflect the unique circumstances of an individual child.

(P14)

*Perceived limitations of ChatGPT compared to personalized professional support:* Another reason for the limited use of ChatGPT was the availability of accessible professional health support and its lack of complete reassurance. Furthermore, mothers emphasized that they felt ChatGPT responses were too general and gave the same answers to everyone. One mother said:

The answers don't really satisfy me; even though they provide temporary relief, I'm not satisfied. It's as if they give the same answer to everyone. On the other hand, when you ask the same question in the hospital, there are different solutions tailored to your situation.

(P5)

A mother whose baby developed a milk allergy in the first month of life further emphasized this perspective. She stated that her child was under special medical supervision due to developing allergies and that she had constant access to her doctor, which reduced the need to resort to alternative sources of information. She said:

Because my child developed allergies, we were under special supervision. What seemed fine to everyone else could be a problem for us, so we had to be in constant communication with someone who knew our situation intimately. Our doctor told us we could reach him at any time of day or night. If we hadn't experienced this allergy, I might not have had this opportunity, and I would rely more on alternative resources like ChatGPT.

(P3)

## 4 Discussion

This qualitative study explored primiparous mothers' experiences of using ChatGPT as a source of health information during early motherhood. The first theme highlights ChatGPT's role as an accessible alternative information source integrated into daily caregiving practices during early motherhood. Primiparous mothers reported turning to ChatGPT primarily due to difficulties in accessing healthcare professionals and a reluctance to seek professional support for concerns perceived as minor or non‐urgent. This finding is consistent with existing literature indicating that mothers frequently experience gaps in professional support after hospital discharge and may hesitate to contact healthcare providers despite ongoing caregiving needs and uncertainties, particularly during the early postpartum period (Duran and Vural 2023; Lupton 2016). In the national context, the absence of a structured nursing‐ or nursery‐based home care system and the lack of postpartum telehealth services often require mothers to contact their primary care physicians directly, which may trigger feelings of hesitation about “disturbing” healthcare professionals. In addition, the demographic profile of the participants, consisting predominantly of mothers in their twenties and thirties, may help explain their strong orientation toward digital information sources. Studies examining digital health information‐seeking behaviors among mothers in similar age groups have reported that millennial mothers are particularly inclined to seek health‐related information through digital platforms (Hikmah 2025; Üstündağ 2024). The widespread digital health information‐seeking behavior observed among millennial mothers appears to be closely associated with their familiarity with technology and their preference for rapid and on‐demand access to information (Setyastuti et al. 2019). These findings should therefore be interpreted within the Turkish healthcare and sociocultural context. Differences in healthcare access, family support structures, digital literacy, and cultural expectations surrounding motherhood may influence how mothers seek information and engage with AI‐based tools. Consequently, mothers' experiences with ChatGPT may differ in settings characterized by different healthcare systems, support structures, or levels of digital access. Therefore, digital tools, when used consciously and within clear boundaries, may provide practical support for low‐risk, routine caregiving concerns, particularly in situations where professional support is not readily accessible.

Our second main theme highlights how mothers perceived ChatGPT as a source of reassurance and emotional comfort during the uncertainties of early motherhood. Another important aspect emphasized by mothers is ChatGPT's non‐judgmental and calming tone. These results are consistent with previous findings showing that repeated access to AI‐assisted, supportive, and guidance‐based information can increase maternal confidence and self‐efficacy, particularly in the context of breastfeeding and early care (Kerimoglu Yildiz et al. 2025; de Vries et al. 2025). Further evidence from evaluations of AI‐powered chatbots for breastfeeding support to expectant mothers shows that ChatGPT increases mothers' confidence in their care decisions (de Vries et al. 2025). These findings suggest that some mothers experienced ChatGPT as a source of reassurance and encouragement during breastfeeding and infant care challenges. They also suggest that ChatGPT may have relational significance for postpartum women. Participants found the tool important not only because it provided quick information, but also because they experienced it as an accessible, responsive, and non‐judgmental tool. This may reflect unmet emotional and communicative needs, particularly in the postpartum period when women may hesitate to seek advice from healthcare professionals or informal support networks. The fact that participants described ChatGPT as a non‐judgmental and readily accessible conversational partner may provide further insight into these experiences. Many mothers reported feeling more comfortable asking questions to ChatGPT than to people in their social circles, suggesting that concerns about being judged, criticized, or perceived as inexperienced may shape help‐seeking behaviors during the transition to motherhood. From this perspective, ChatGPT's appeal may stem not only from the information it provides but also from the absence of social evaluation. Rather than reflecting a preference for AI over human interaction, these experiences may highlight unmet relational and communicative needs in both formal healthcare encounters and informal support networks. Previous research has shown that AI chatbots can be perceived as empathetic in text‐based health‐related interactions (Howcroft et al. 2025); however, this perceived empathy should be interpreted cautiously, as AI‐mediated responses cannot fully replace the relational, contextual, and tangible dimensions of human support and professional care (Rice et al. 2021; Ayers et al. 2023). While participants often described feeling reassured after interacting with ChatGPT and perceived it as helpful in managing uncertainty, its use may also influence help‐seeking practices and alter how mothers engage with traditional sources of support. This raises important questions regarding whether AI‐mediated support primarily complements existing interpersonal support systems or, in some circumstances, partially substitutes interactions that might otherwise occur with healthcare professionals, family members, or peers. Therefore, the use of ChatGPT in early motherhood should be considered not only as a digital information quest but also as an emotional and relational phenomenon. However, while many mothers find comfort in using ChatGPT, its benefits become most apparent when used within conscious and clear boundaries. Limited use can provide reassurance, offer practical solutions to daily care challenges, and help mothers cope with uncertainty in situations where immediate professional support is not needed.

Despite the widespread use of ChatGPT, the third theme highlights that primiparous mothers established clear boundaries regarding the role of this tool in caregiving. Participants described using ChatGPT primarily for routine breastfeeding and infant care questions while preferring professional healthcare support when faced with serious, uncertain, or medically complex situations. This selective use suggests that mothers did not place unconditional trust in AI‐generated information; rather, they consciously evaluated when digital support was appropriate and when professional expertise was required. A similar pattern was reported by Üstündağ (2024), who found that although millennial mothers frequently used digital resources, their digital awareness was reflected in their ability to recognize potential risks, establish boundaries, and make informed decisions about the use of technology in caregiving. Our findings extend this observation by suggesting that mothers viewed ChatGPT as a useful source of general information but recognized its limitations in situations requiring individualized assessment and clinical judgment. These perceptions are consistent with broader concerns regarding the reliability, contextual sensitivity, and clinical appropriateness of AI‐generated health information (Byrd et al. 2025; de Vries et al. 2025). Unlike healthcare professionals, generative AI systems cannot perform physical assessments, evaluate the full clinical context, or tailor recommendations to an infant's unique medical history and evolving health status. Furthermore, AI‐generated responses may occasionally contain inaccuracies or “hallucinations,” in which incorrect information is presented with apparent confidence (Giuffrè et al. 2024). Such limitations may be particularly consequential in infant health, where subtle differences in symptoms or risk factors can substantially influence clinical decision‐making. Consistent with previous literature, these concerns highlight the potential risks of delayed diagnosis, postponed healthcare seeking, or inappropriate caregiving decisions when AI‐generated information is relied upon without professional consultation (Ayre et al. 2024). Thus, these findings suggest that ChatGPT may be used by some mothers as an additional source of information for low‐risk and routine caregiving concerns; however, it cannot substitute for individualized clinical assessment and professional healthcare support.

### 4.1 Limitations

This study has some limitations. First, the sample consisted only of first‐time mothers; this may limit the transferability of the findings to multiple mothers who may have different caregiving experiences and information‐seeking behaviors. Second, the age and educational level range of the participants is relatively narrow, with the majority of mothers belonging to the millennial generation and high educational level. Since digital literacy, attitudes toward technology, and trust in AI‐based tools can differ across generations and educational levels, the findings may not fully reflect the experiences of all age and educational level groups. Third, participants were recruited from a single university hospital in Türkiye through routine pediatric follow‐up services. As such, characteristics such as marital status, mode of delivery, age at first pregnancy, access to healthcare, family support structures, and use of digital technologies may reflect common features of first‐time mothers receiving care within this healthcare context. These factors may have influenced participants' access to professional support, their information‐seeking behaviors, and their use of ChatGPT as an additional source of information. Furthermore, cultural expectations surrounding motherhood and childcare may have shaped how participants perceived and used AI‐based information tools. Therefore, the findings are most transferable to settings with similar sociocultural, healthcare, and digital access characteristics.

## 5 Conclusion

The findings indicate that primiparous mothers primarily perceive ChatGPT as an accessible and supportive source of health information that helps them manage uncertainty, reassurance during moments of concern, and strengthen confidence in everyday decision‐making during early motherhood. At the same time, mothers demonstrated awareness of ChatGPT's limitations, preferring individualized assessment and professional guidance in such situations. Overall, ChatGPT may serve as a valuable supplementary tool in the postpartum period by offering timely information and reassurance; however, it cannot replace personalized professional care. Future research should examine in greater depth how mothers evaluate AI‐based health tools, the conditions under which trust in these tools increases or decreases, and how informed and safe use can be supported. In parallel, healthcare professionals have an important role in acknowledging mothers' use of digital resources and providing guidance on appropriate, safe, and evidence‐informed use, so that AI‐based tools can be integrated into early motherhood as supportive yet clearly bounded resources.

## Author Contributions

E.H.S., M.N.T., E.H.K.C., P.G.G., designed the research study. E.H.S. performed the research and conducted the in‐depth interviews. E.H.S., M.N.T., E.H.K.C., P.G.G., and N.D.K. contributed to the data management and organization using MAXQDA software. E.H.S. led the data analysis, while M.N.T., E.H.K.C., P.G.G., and N.D.K. participated in the reflexive thematic analysis and the refinement of emerging themes. E.H.S., M.N.T., E.H.K.C., P.G.G., and N.D.K. drafted and critically revised the manuscript. All authors have read and approved the final manuscript.

## Funding

The authors received no funding for this work.

## Conflicts of Interest

The authors declare no conflicts of interest.

## Data Availability

The data that support the findings of this study are available on request from the corresponding author. The data are not publicly available due to privacy or ethical restrictions.
